# Green and Abrasion-Resistant Superhydrophobic Coatings Constructed with Tung Oil/Carnauba Wax/Silica for Wood Surface

**DOI:** 10.3390/ma17123000

**Published:** 2024-06-19

**Authors:** Jieying Su, Haitao Zhang, Meiting Zhu, Jiajie Cai, Bin Xu

**Affiliations:** College of Materials Science and Engineering, Zhejiang University of Technology, Hangzhou 310014, China; nirvana7s@163.com (J.S.); 15525092503@163.com (H.Z.); 13395621572@163.com (M.Z.); caijiajie1133@163.com (J.C.)

**Keywords:** superhydrophobic, carnauba wax, tung oil, silica nanoparticles, abrasion resistant, transparent

## Abstract

As a renewable, environmentally friendly, natural, and organic material, wood has been receiving extensive attention from various industries. However, the hydrophilicity of wood significantly impacts the stability and durability of its products, which can be effectively addressed by constructing superhydrophobic coatings on the surface of wood. In this study, tung oil, carnauba wax, and silica nanoparticles were used to construct superhydrophobic coatings on hydrophilic wood surfaces by a facile two-step dip-coating method. The surface wettability and morphology of the coatings were analyzed by a contact angle meter and scanning electron microscope, respectively. The results suggest that the coating has a micron–nanosized two-tiered structure, and the contact angle of the coating is higher than 150° and the roll-off angle is lower than 10°. Sandpaper abrasion tests and UV diffuse reflectance spectra indicate that the coatings have excellent abrasion resistance and good transparency. In addition, the coated wood shows excellent self-cleaning and water resistance, which have great potential for applications in industry and furniture manufacturing.

## 1. Introduction

Wood, as an abundantly available renewable resource, due to its advantages of being lightweight and beautiful, has been widely used in building materials, furniture manufacturing, and interior decoration. However, the hydrophilic property of wood causes it to be susceptible to deterioration and dimensional instability, which significantly limits its scope of use [1,2]. An effective way to avoid the above problems is to modify wood to introduce superhydrophobicity, which can isolate direct water contact [3]. Lin et al. produced dimensionally and thermally stable antifouling wood by modifying the wood with poly(methylhydrogen)siloxane, which had a contact angle of 150° in the cross-section of the wood [4]. Ou et al. produced a superhydrophobic coating on the wood surface by one-step immersion in a composite silane solution, which provided the wood with excellent water/moisture repellency [5]. Cao et al. constructed a transparent superhydrophobic coating by brushing a ZIF-8/HDTMS hybrid emulsion on the wood surface, which improved the stain resistance and durability of the wood’s surface [6].

Superhydrophobic surfaces typically exhibit extreme water repellency, possessing high contact angles (>150°) and low roll-off angles (<10°) [7,8]. Superhydrophobic coatings have been widely used in applications such as self-cleaning [9], corrosion protection [10], anti-icing [11], and water–oil separation [12]. In recent years, numerous methods to construct superhydrophobic coatings on wood surfaces have been proposed. Wang et al. prepared a superhydrophobic surface on wood by preparing silica coating through a sol–gel process and fluorination [13]. In order to satisfy the demands of esthetics, the coating has to be as transparent as possible to retain the color and grain of the original wood while achieving superhydrophobic properties. Yue et al. fabricated a transparent and durable polysiloxane/SiO_2_ coating for wood surfaces [14]. In addition, low-surface-energy reagents such as fluorine-containing and petroleum-based long-chain hydrocarbons are often used to prepare superhydrophobic coatings. Nevertheless, these reagents show difficulty degrading in the ecosystem, which can cause environmental and health problems [15,16,17]. Natural waxes, such as carnauba wax and beeswax, provide an environmentally friendly source of low-surface-energy hydrocarbons for superhydrophobic coatings, which are extremely abundant in nature [18,19]. Zhang et al. constructed a superhydrophobic surface with a low roll-off angle on paper by coating with a mixed emulsion of beeswax and carnauba wax [20].

However, pure wax-based superhydrophobic coatings still have poor abrasion resistance due to the low toughness of carnauba wax, which results in weak bonding to a substrate when used alone. The poor abrasion resistance of pure wax-based superhydrophobic coatings limits the practical application of superhydrophobic coatings [21,22,23]. In recent years, extensive attention has been directed towards increasing the adhesion strength between coatings and wood substrates [24,25]. Liu et al. fabricated a PVA/SiO_2_ coating on a wooden substrate by drop-coating, which improved the mechanical robustness of the superhydrophobic surface [26]. Li et al. prepared a superhydrophobic material with excellent durability by adding epoxy resin as a binder [27]. Mastouri et al. constructed wax-based superhydrophobic coatings with PDMS and epoxy resin, respectively [28]. The results indicate that layered structures created by wax self-assembly and interwoven morphology can facilitate the stability of coatings.

Tung oil is extracted from the seeds or kernels of the tung tree, and mainly consists of α-elaeostearic acid, oleic acid, and linolenic acid [29]. Tung oil is also an environmentally friendly, low-surface-energy material, which is often used for wood protection. Compared to other vegetable oils, tung oil can quickly dry without desiccant [30]. The autoxidation process of tung oil is a free radical chain reaction. During the natural drying process, the tung oil is cured by oxidative polymerization and free radical polymerization under air and light [31,32]. The curing of the tung oil is able to form a solidified film on the internal surface layer of the wood, which provides a higher mechanical stability and temperature stability.

Pinus wood is widely used in furniture manufacturing because of its simple, clear, and original grain pattern and desirable texture. However, pinus wood is easily discolored when exposed to moisture, which can impair the esthetics. We chose pinus wood as the substrate to study a transparent superhydrophobic coating that protects the pinus wood from moisture.

This study has developed a simple method to prepare superhydrophobic coatings with excellent abrasion resistance and transparency on wood surfaces. Tung oil/carnauba wax was used as the base layer to construct a tung oil/carnauba wax/silica superhydrophobic composite coating by a two-step impregnation method. The addition of tung oil can regulate the brittleness of carnauba wax. Tung oil is cured by oxidative polymerization and free radical polymerization on the internal surface of the wood, which strengthens the bond between the tung oil/carnauba wax network and the wood substrate, improving the abrasion resistance. In conclusion, this study will provide a simple, efficient, and green method to prepare superhydrophobic coatings with high abrasion resistance.

## 2. Materials and Methods

### 2.1. Materials

Pinus wood was obtained from Suqian Xinyi Co., Ltd. (Suqian, China) with a density of 0.45 g/cm^3^. The pinus wood was cut into 3 cm (longitudinal) × 3 cm (tangential) × 1 cm (radial) blocks and sanded along the longitudinal surface with 400-mesh sandpaper. The wood was washed with deionized water and ethanol to remove dust and wood shavings on the surface of the wood, and then dried in an oven at 60 °C. All experiments used wood that had been washed and dried as mentioned above.

Carnauba wax was obtained from Guangzhou Longstone Co., Ltd. (Guangzhou, China) Tung oil was obtained from Ta-pieh Mountains Tung Oil Workshop Co., Ltd. (Xinyang, China) Sodium chloride and ethyl acetate were provided by Sinopharm Chemical Reagent Co., Ltd. (Shanghai, China) Hydrophobic (lipophilic) nano-SiO_2_ was purchased from NANO Technology Co., Ltd. (Shaoxing, China) All the nano-SiO_2_ used in the experiments was hydrophobic and lipophilic. Ethanol was obtained from Hangzhou Shuangmu Chemical Co., Ltd. (Hangzhou, China) Sodium hydroxide and hydrochloric acid were purchased from Shanghai Aladdin Biochemical Technology Co., Ltd. (Shanghai, China).

### 2.2. Preparation of Superhydrophobic Composite Coatings

**Figure 1:**
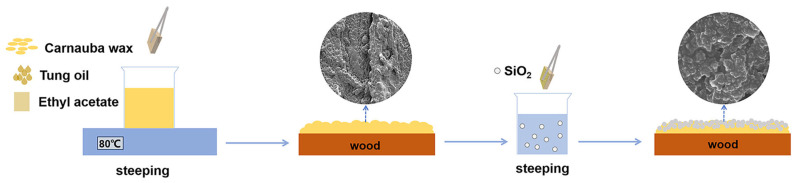
Preparation process of superhydrophobic composite coating with tung oil/carnauba wax/silica.

The preparation process is shown in Figure 1. Different ratios (Table 1) of carnauba wax and tung oil were added into 45 g of ethyl acetate, followed by heating and stirring in a water bath at 80 °C until the carnauba wax and tung oil were completely dissolved. A total of 5 g of hydrophobic nano-SiO_2_ was added to 45 g of ethanol and ultrasonically dispersed for 10 min to obtain a 10% hydrophobic nano-SiO_2_ dispersion. The wood was vertically immersed in the tung oil/carnauba wax mixture for 30 s and then lifted out of the mixture. Subsequently, the wood was rapidly immersed in the sonicated hydrophobic nano-SiO_2_ dispersion for 10 min, and then picked up. Wood with a superhydrophobic composite coating was obtained by drying in a fuming cupboard for 7–8 days. Except for the transparency experiments, the average SiO_2_ particle size of the samples used in the other experiments was 80 nm.

### 2.3. Characterization

The contact angles and sliding angles of all samples were measured by a contact angle meter (OCA30, Dataphysics, Filderstadt, Germany). The contact angle and sliding angle were measured with 4 μL and 10 μL of water droplets at three different locations, respectively, and the final test results were the arithmetic mean of these three measurements. The surface morphology of samples was observed by scanning electron microscopy (SEM, Regulus 8100, Hitachi, Tokyo, Japan). The surfaces of the samples were sprayed with gold before imaging. The diffuse reflectance spectra of the samples in the visible range (400–800 nm) were analyzed by a UV-Vis spectrophotometer (Lambda750, PerkinElmer, Waltham, MA, USA).

The abrasion resistance of the coating was investigated by a sandpaper abrasion test according to [33]. The wood was placed on a flat sheet of 800-mesh sandpaper. Subsequently, the wood was pushed under the condition that a 100 g weight was placed in the center of the wood. The single-directional distance of the pushing was 10 cm, one round trip was one cycle, and three positions were selected for a contact angle measurement after five cycles of abrasion. The final results were the arithmetic mean of these three measurements.

The chemical stability of the coatings was tested by the chemical liquid immersion method. The samples were placed in three different solutions of sodium chloride (3 wt%), sodium hydroxide (pH = 13), and hydrochloric acid (pH = 1) and left to stand for 0–24 h. Three different positions were selected to measure the contact angle. The final results were also the arithmetic mean of these three measurements.

The dust was sprinkled on the surface of the samples with a certain inclination angle. Subsequently, a small amount of water was used to rinse the surface and the cleanliness of the sample surface was observed.

The uncoated and coated wood samples were immersed in deionized water to investigate the effect of the superhydrophobic coating on the water resistance of the wood. The wood was removed after every interval and the weight was recorded by absorbing the surface moisture with filter paper. The water uptake (*WU*) was calculated from the change in weight of the samples before and after impregnation by Equation (1):

(1) $$WU\left(\%\right)=\frac{W_{2}-W_{1}}{W_{1}}\times 100$$

where *W*_1_ is the weight before water absorption and *W*_2_ is the weight after water absorption. 

## 3. Results

### 3.1. Morphology and Wettability

**Figure 2:**
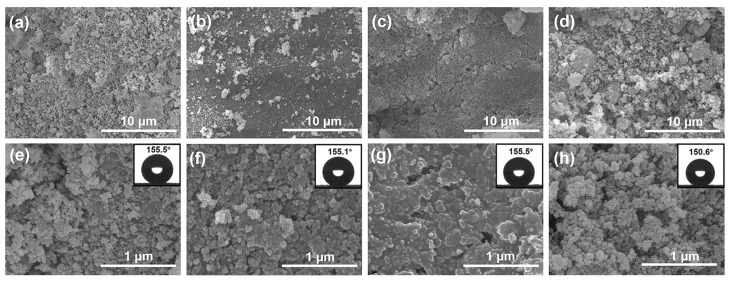
SEM and water contact angle (inset) images of (**a**,**e**) W1T0, (**b**,**f**) W1T1, (**c**,**g**) W1T4, and (**d**,**h**) W0T1.

**Figure 3:**
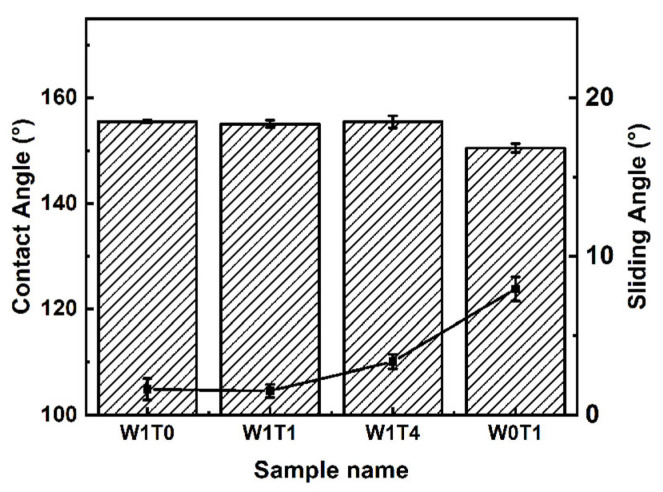
Contact angle and sliding angle of composite coatings.

The successful synthesis of a superhydrophobic composite coating was confirmed by characterizing morphology and wettability. Figure 2 shows the panoramic and magnifying SEM micrographs of W1T0, W1T1, W1T4, and W0T1, and the corresponding contact angle image is exhibited in the upper right corner of the magnifying SEM micrograph. The micrographs of W1T0, W1T1, and W1T4 show that the nano-SiO_2_ adheres to the surface of the tung oil/carnauba wax base layer, and some of the nano-SiO_2_ is embedded in the base layer. As the ratio of tung oil to carnauba wax dosage increases, the embedding of SiO_2_ becomes more obvious. The appearance of this phenomenon may be related to the addition of tung oil. The wax cools and crystallizes to form a crystalline network which can capture the tung oil to form a semi-solid substance [34]. The nano-SiO_2_ gradually sinks into the coating before the wax/oil network hardens completely. The W1T4 surface has a micron–nano-sized two-tiered structure, with nano bumps composed of nano-SiO_2_ on top of the micron bumps composed of carnauba wax as shown in Figure 2c,g. This binary structure is able to capture more air and has a higher water repellency compared to a single microscale structure [35]. The nano-SiO_2_ in the upper layer of W0T1 is just physical aggregation and the base layer is invisible, as depicted in Figure 2d,h. The contact angle insets of the SEM micrograph show the excellent hydrophobicity of the coating, with contact angles all greater than 150°. In addition, the sliding angles of all samples are less than 10°, as shown in Figure 3.

### 3.2. Abrasion Resistance Analysis 

**Figure 4:**
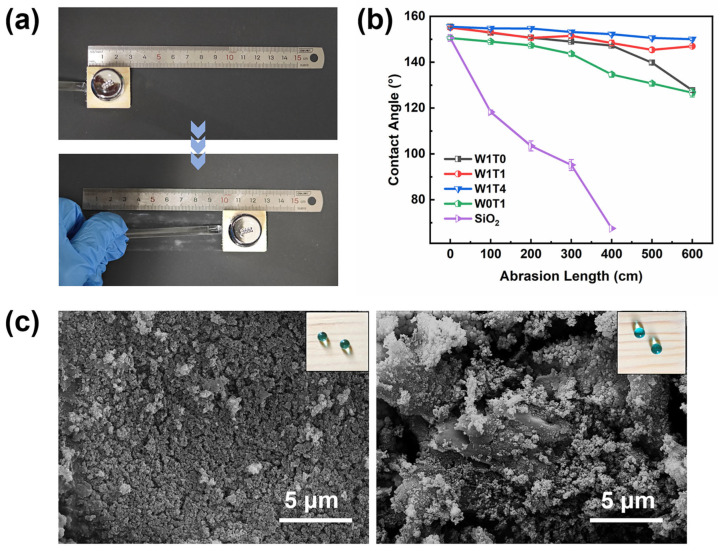
(**a**) Sandpaper abrasion test. (**b**) Change in contact angle of the coating with abrasion distance. (**c**) Morphology of W1T4 before and after abrasion and digital photo of water droplets on the surface (inset).

Sandpaper abrasion tests were carried out with the equipment shown in Figure 4a. The non-base-layer-containing sample prepared by the impregnation method was used as a control. Figure 4b shows that the decrease in contact angle of the non-base-layer-containing sample is significantly higher than the samples with a base layer. The contact angle of the non-base-layer-containing sample was similar to the original wood after 400 cm of abrasion. The W1T0 and W0T1 samples show a significant increase in abrasion resistance compared to the control group. This increase is attributed to the use of carnauba wax or tung oil as binders that help to improve the adhesion strength between the coating and the substrate. However, they are still inferior in terms of abrasion resistance compared to W1T1 and W1T4, which have rough structures composed of micron-sized bumps of wax and nano bumps of SiO_2_. The W1T4 sample retains excellent superhydrophobic performance after 600 cm of abrasion, and the contact angle has the lowest decrease of all the samples, from 155.5 ± 0.8° to 150 ± 0.5°. The superior abrasion resistance of W1T4 is due to two main aspects. The SiO_2_ nanoparticles are located between the wax bumps and protected by the tung oil/carnauba wax network during the abrasion process. On the other hand, the tung oil infiltrated into the carnauba wax and wood, which can strengthen the bond between the coating and wood by curing after autoxidation. The hydrogen on the α-C in tung oil was oxidized and then reacted with oxygen molecules to form free radicals and peroxyl radicals. These free radicals can polymerize with each other and trigger the polymerization of other tung oil molecules further. Figure 4c shows the morphology of W1T4 before and after abrasion. The coating surface before abrasion was covered with a dense nano-SiO_2_ layer, and the nano-SiO_2_ was firmly adhered by the tung oil and carnauba wax base layers. After abrasion, the coating loses some SiO_2_ and exposes part of the base layer structure. However, the coating still has superhydrophobic performance. The inset in the SEM micrograph shows digital photos of water droplets on the surface of W1T4 before and after abrasion, showing that the water droplets still have a bead shape on the sample surface after abrasion. The results indicate that the superhydrophobic composite coating has excellent abrasion resistance. The tung oil/carnauba wax/silica hydrophobic composite coating shows advantages in abrasion resistance compared to the other reported coatings in Table 2.

### 3.3. Chemical Stability Analysis

**Figure 5:**
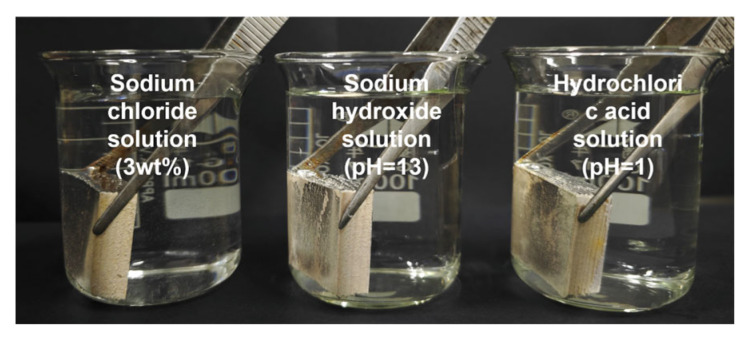
Digital photo of coated wood immersed in sodium chloride solution (3 wt%), sodium hydroxide solution (pH = 13), and hydrochloric acid solution (pH = 1).

Digital photos of the coated woods immersed in the sodium chloride solution (3 wt%), sodium hydroxide solution (pH = 13), and hydrochloric acid solution (pH = 1) are shown in Figure 5. Sodium hydroxide immersion has the most significant effect on the hydrophobic properties of coated wood, as shown in Table 3, since fatty acid esters are the main component of carnauba wax, and tung oil also has a high content of triglyceride, which results in the saponification of the coating in a sodium hydroxide solution [39]. The coated wood loses its superhydrophobic properties when immersed in sodium hydroxide solution for 1 h. The coating is seriously damaged after 4 h of immersion and the wood surface becomes hydrophilic. The sodium chloride solution has little effect on the coating. The coated wood still has a contact angle of 152.7° after 24 h immersion in the sodium chloride solution. The superhydrophobic property of the coating decreased slightly when immersed in the acid solution, and the contact angle of the coating was maintained at 150.2° after 12 h of immersion. The results indicate that the coating has excellent stability in sodium chloride and hydrochloric acid solutions, but alkaline solutions can destroy the coating.

### 3.4. Transparency Analysis 

**Figure 6:**
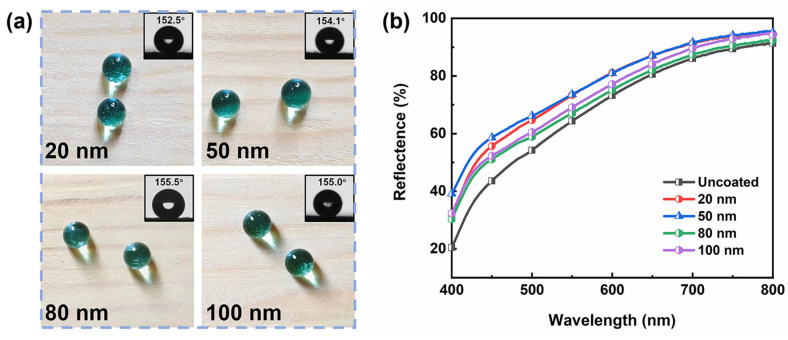
(**a**) Digital photos, contact angles (inset), and (**b**) diffuse reflectance spectra of composite coatings prepared by hydrophobic nano-SiO_2_ with different particle sizes.

Different particle sizes of hydrophobic SiO_2_ (average particle sizes of about 20 nm, 50 nm, 80 nm, and 100 nm) were used to prepare composite coatings of tung oil/carnauba wax/silica (based on the W1T4 sample) on the wood surface. Figure 6a shows that the coating contact angles are all higher than 150° and the grain of the wood is preserved. A significant increase in the reflectance of coated woods in the blue-green part of the visible spectrum is observed by comparing the diffuse reflectance spectra of the uncoated and coated woods in Figure 6b, which is attributed to an increase in the Rayleigh scattering cross-section (~λ^−4^) [40]. The increased reflectivity reduces the saturation of the wood, resulting in a slight whitening of the surface. The coating prepared by 80 nm hydrophobic SiO_2_ particles has the closest reflectance to uncoated wood, indicating that the samples prepared by 80 nm SiO_2_ have the best transparency.

### 3.5. Self-Cleaning Properties

**Figure 7:**
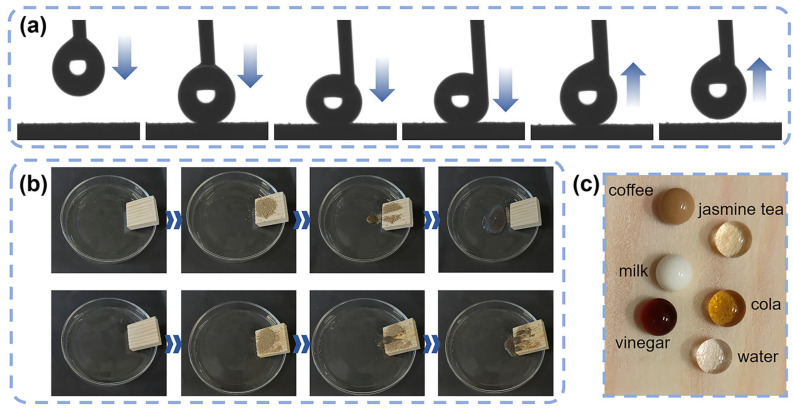
(**a**) Process of water droplets being squeezed and lifted from the composite-coated surface. (**b**) Process of water droplets rolling off the surface of coated and uncoated wood (the states change along the arrows). (**c**) Digital photo of several common liquids (tea, cola, water, coffee, milk, vinegar) on the composite-coated surface.

Figure 7a shows the process of slowly applying 4 μL of water onto the coated surface and lifting the dropper up when the droplet was squeezed to deformation. In addition, the arrows indicate the direction of water droplet movement in Figure 7a. The coating surface has no liquid residue, indicating that the adhesion of the coating surface to water is extremely low. The water droplets roll off like beads on the surface of the coating while carrying away the soil, as shown in Figure 7b. As the water dripped, all soil on the surface of the coating was carried away, and the surface of the wood was restored to a clean state. This result is due to the low adhesion of the coating surface to water, and the soil is attracted and carried away by the water droplets rolling on the coating surface. The uncoated wood is hydrophilic. Droplets of water flow and mix with the soil. Wet soil can stick to the wood, resulting in a dirtier surface. Wood with coatings is also repulsive to common household liquids such as tea, cola, water, coffee, milk, and vinegar, as shown in Figure 7c. The results show that the coating has excellent self-cleaning ability.

### 3.6. Water Absorptions

**Figure 8:**
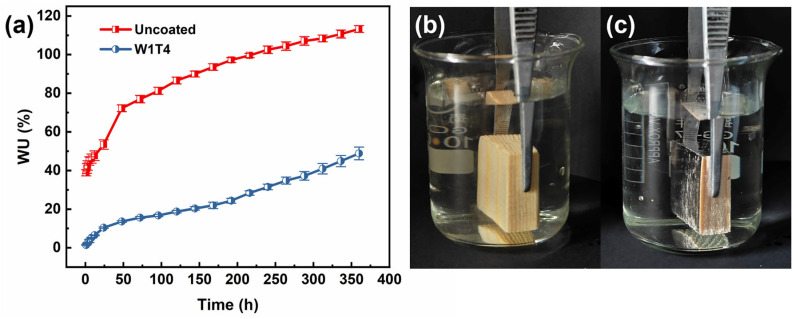
(**a**) Change in the water absorption of the original and modified wood as a function of the immersion time in water. Immersion of (**b**) uncoated wood and (**c**) W1T4 in water.

Figure 8a shows that the process of water absorption in uncoated wood can be divided into two stages. The first stage is 0–48 h. The mass of the uncoated wood increased significantly after water absorption. This result is due to the large number of pores contained within the wood. The pores lead to a high water contact area and low resistance to penetration, allowing water to infiltrate rapidly. The second stage is 48–360 h, and during this stage the mass increase rate of the uncoated wood is slowed down. The decrease in the rate is attributed to the fact that the water absorption is nearly saturated around the periphery of the wood, and the water infiltrates mainly in the center at this time. Compared to the first stage, the water contact area is reduced, and infiltration resistance is increased, resulting in slower water infiltration.

The change in mass of the coated wood during the water absorption process is significantly different from the uncoated wood. The water absorption process of the coated wood is also divided into two stages. The first stage is 0–192 h, and the mass of the coated wood changes slowly during this stage. The mass of the coated wood increased by 24.37% at 192 h, which is significantly less than the mass increase in the uncoated wood immersed for 1 h. This disparity is attributed to the formation of an air film by the micro–nano structure of the superhydrophobic coating. This air film effectively isolates water from direct contact with the wood, leading to a slow increase in the mass of the coated wood. The second stage is 192–360 h, and the mass increase rate of the coated wood is higher than the previous stage. This higher mass increase rate is attributed to the process in which part of the water breaks through the air film and touches the recessed portion of the coating. This process is typical of the transition from the Cassie–Baxter model to the Wenzel model, resulting in the mass increase rate of the samples rising [41].

After 360 h of testing, the mass of the coated wood increased by 48.84%, and the mass of the uncoated wood increased by 113.25%. The mass increase rate of the coated wood decreased by 56.87% compared to the uncoated wood. This result indicates that the application of superhydrophobic coatings can significantly reduce the water absorption of wood.

In order to visualize the formation of air pockets on the surface of W1T4, uncoated wood and W1T4 were immersed in water separately. Figure 8b shows the uncoated wood exhibiting clear wood grain in water. Figure 8c shows a bright silver mirror appearing on the surface of W1T4. This specularity is produced by the total reflection of light, owing to the air trapped by the micro–nano structures of the coating [42,43]. Since the Cassie–Baxter model assumes that the droplets are located at the top of the micro–nano structure, the water droplets stay on the composite gas–solid interface. The bright silver mirror phenomenon indicates that the wetting state of the superhydrophobic coating is the Cassie–Baxter state. The contact state of water with the coating transitioned from Cassie–Baxter to Wenzel when immersed in water. This transition is due to the penetration of liquid droplets into the interstices of the micro–nano structures when the Laplace pressure at the air–liquid interface overpowers the capillary forces [44]. The superhydrophobic composite coating of tung oil/carnauba wax/silica is compared with other coatings for water absorption as shown in Table 4. The water absorption of the coated wood is significantly less than that shown in the other reported works after 120 h of immersion, demonstrating that the tung oil/carnauba wax/silica superhydrophobic composite coating has excellent water resistance properties.

## 4. Conclusions

In this study, an abrasion-resistant and transparent superhydrophobic coating was constructed on the wood surface with tung oil/carnauba wax as the substrate, and hydrophobic nano-SiO_2_ was introduced for the construction of micro–nano structures by a simple impregnation method. In addition, the effects of different substrate ratios and hydrophobic nano-SiO_2_ particle sizes on the performance of superhydrophobic coatings were investigated. The results suggest that the coating with a ratio of tung oil/carnauba wax at 1:4 has the optimal abrasion resistance, which still had a contact angle of over 150° after 600 cm of abrasion. At this ratio, the coating prepared by hydrophobic nano-SiO_2_ with a particle size of 80 nm has optimal transparency and basically preserves the grain of the original wood. The results of our self-cleaning experiments indicate that the composite coating has excellent self-cleaning properties and can be widely used in the fields of furniture manufacturing and interior decoration. Furthermore, water resistance tests have demonstrated that the coating has excellent water resistance, which can maintain the stability of the wood in practical applications.

## Figures and Tables

**Table 1:** Ratios of tung oil/carnauba wax mixture solution.

Sample	Carnauba Wax (g)	Tung Oil (g)
W1T0	5	0
W1T1	2.5	2.5
W1T4	1	4
W0T1	0	5

**Table 2:** Abrasion resistance of other superhydrophobic coatings compared with tung oil/carnauba wax/silica composite coatings.

Coating	Fabrication Technique	Contact Angle (°)	Weight (g)	Sandpaper (mesh)	Abrasion Length/Cycles	Ref.
PDMS @ stearic acid-Al(OH)_3_	Dip and deposition coating	156.0	200	1500	240 cm abrasion length	[36]
ZIF-8/HDTMS	Brush coating	153.0	50	1000	10 abrasion cycles (100 cm)	[6]
Silica/epoxy resin/FAS	Dip coating	152.0	200	1500	250 cm abrasion length	[37]
CNF/PDMS	Spray coating	158.0	100	800	19 abrasion cycles (380 cm)	[38]
Tung oil/carnauba wax/silica	Dip coating	155.5	100	800	600 cm abrasion length	This work

**Table 3:** Contact angles of coated wood after immersion in sodium chloride solution (3 wt%), sodium hydroxide solution (pH = 13), and hydrochloric acid solution (pH = 1) for a period of time.

Solution	Time (h)	Contact Angle (°)
Sodium chloride solution (3 wt%)	24	152.7
Sodium hydroxide solution (pH = 13)	1	140.9
Sodium hydroxide solution (pH = 13)	4	79.3
Hydrochloric acid solution (pH = 1)	12	150.2

**Table 4:** Water absorption of other superhydrophobic coatings compared with tung oil/carnauba wax/silica composite coatings.

Base Material	Coating	Fabrication Technique	Time (h)	Uncoated (WU%)	Coated (WU%)	Ref.
Cunninghamia lanceolata	SiO_2_/PMHS ORMOSIL	Dip coating	24	70.30	24.10	[45]
Pinus wood	Epoxy/Cu_2_(OH)_3_Cl NPs/stearic acid	In situ growth and Dip coating	120	80.00	36.00	[46]
Cunninghamia lanceolata	Ammonia–HMDS	Vapor treatment coating	24	72.30	31.90	[47]
Pinus wood	Tung oil/carnauba wax/silica	Dip coating	120	86.61	18.72	This work
Pinus wood	Tung oil/carnauba wax/silica	Dip coating	360	113.25	48.84	This work

## Data Availability

The original contributions presented in the study are included in the article, further inquiries can be directed to the corresponding author.
